# Regulating Ru–Ru Distance in RuO_2_ Catalyst by Lattice Hydroxyl for Efficient Water Oxidation

**DOI:** 10.1021/acsnano.5c01937

**Published:** 2025-05-06

**Authors:** Sixuan She, Hsiao-Chien Chen, Changsheng Chen, Yanping Zhu, Gao Chen, Yufei Song, Yiping Xiao, Zezhou Lin, Di Zu, Luwei Peng, Hao Li, Ye Zhu, Yuen Hong Tsang, Haitao Huang

**Affiliations:** † Department of Applied Physics, 26680 The Hong Kong Polytechnic University, Hung Hom, Kowloon, Hong Kong, China; ‡ Center for Reliability Science and Technologies, 56081 Chang Gung University, Taoyuan 33302, Taiwan; § Kidney Research Center, Department of Nephrology, Chang Gung Memorial Hospital Linkou, Taoyuan 33305, Taiwan; ∥ Jiangsu Key Laboratory of New Energy Devices and Interface Science, School of Chemistry and Materials Science, Nanjing University of Information Science and Technology, Nanjing 210044, China; ⊥ School of Materials Science and Engineering, Beijing Institute of Technology, Beijing 100081, China; # Photonics Research Institute, The Hong Kong Polytechnic University, Hung Hom, Kowloon, Hong Kong, China; ∇ Research Institute for Advanced Manufacturing, The Hong Kong Polytechnic University, Hung Hom, Kowloon, Hong Kong, China

**Keywords:** lattice hydroxyl, oxygen evolution reaction, proton exchange membrane water electrocatalysis, Ru−Ru distance, ruthenium oxide

## Abstract

Highly active and durable electrocatalysts for the oxygen evolution reaction (OER) are crucial for proton exchange membrane water electrolysis (PEMWE). While doped RuO_2_ catalysts demonstrate good activity and stability, the presence of dopants limits the number of exposed active sites and complicates Ru recovery. Here, we present a monometallic RuO_2_ (d-RuO_2_) with lattice hydroxyl in the periodic structure as a high-performance OER electrocatalyst. The obtained d-RuO_2_ catalyst exhibits a low overpotential of 150 mV and long-term operational stability of 500 h at 10 mA cm^–2^, outperforming many Ru/Ir-based oxides ever reported. A PEMWE device using d-RuO_2_ sustains operation for 348 h at 200 mA cm^–2^. In-situ characterization reveals that the incorporation of lattice hydroxyl increases the Ru–Ru distance, which facilitates the turnover of the Ru oxidation state and promotes the formation of stable edge-sharing [RuO_6_] octahedra during the OER, thereby accelerating the formation of O–O bonds and suppressing the overoxidation of Ru sites. Additionally, the small particle size of the catalyst decreases the three-phase contact line and promotes bubble release. This study will provide insights into the design and optimization of catalysts for various electrochemical reactions.

## Graphical abstract

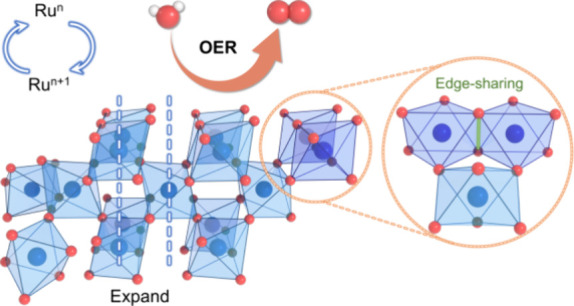

## 1 Introduction

The conversion of renewable electricity into hydrogen offers a promising pathway for the utilization of renewable energy sources, such as solar, wind, and tidal energies.^1^ Proton exchange membrane water electrolysis (PEMWE) plays a crucial role in this scenario, thanks to its ability to quickly adapt to fluctuations in renewable electricity supply.^2^ The widespread deployment of PEMWE is primarily constrained by the anodic oxygen evolution reaction (OER), because (i) the sluggish four-electron reaction process requires significant energy input, and (ii) the acidic environment, high oxidation potential, and oxygen-rich atmosphere limit the selection of catalysts.^3,4^ Currently, iridium-based catalysts are the predominant choice for commercial use due to their good dissolution resistance.^5–7^ Given the current iridium demand of 67 t GW^–1^, the iridium production rate of 7 t a^–1^ cannot meet the target installation capacity of 61 GW by 2045.^8^ Therefore, iridium-free OER electrocatalysts with high catalytic activity and stability are critical to the large-scale application of PEMWE.

As low-cost alternatives to IrO_2_, RuO_2_-based electrocatalysts have garnered considerable attention due to their good OER activity.^9–12^ Recent work by Chorkendorf and co-workers pointed out that less than 0.2% of the released oxygen contains oxygen atoms originating from RuO_2_ itself, suggesting that RuO_2_ predominantly follows the adsorbate evolution mechanism (AEM) or the oxide path mechanism (OPM), rather than the lattice oxygen mechanism (LOM).^13,14^ In the AEM and OPM, the strong adsorption of oxygen-containing species on the RuO_2_ surface affects the formation of O–O bonds, limiting the catalytic efficiency.^15–18^ Moreover, RuO_2_ easily transforms into soluble RuO_4_ under high oxidation potentials, leading to structural collapse and rapid dissolution of Ru sites.^19,20^ To overcome these limitations, heterometal elements are often required to modulate the electronic and crystal structure of RuO_2_.^21–24^ For instance, Guo and co-workers proposed that introducing lanthanide elements with 4f orbitals into RuO_2_ precisely tunes the Ru–O covalency, which reduces the adsorption of oxygen-containing species and enhances the formation energy of lattice oxygen and Ru vacancy.^21^ Qiao and co-workers incorporated the electron donor Re to induce the formation of high-valence reactive Ru sites and facilitate charge transfer during the OER.^22^ While heterometal elements enhance catalytic performance, they reduce the number of exposed Ru active sites and complicate Ru recovery, which restricts their commercial potential. Thus, alternative strategies are highly desirable to improve the activity and stability of monometallic RuO_2_.

Surface reconstruction is common under OER operating conditions, but it often leads to the formation of amorphous or poorly crystalline structures, which compromise structural stability.^25–27^ Only a few illustrations have shown that reconstruction brings out stable configurations.^28,29^ In the perovskite (ABO_3_) system, for example, the dissolution of A-site ions facilitates the transformation of corner-sharing [BO_6_] octahedra into a more stable edge-sharing arrangement.^28^ Compared with corner-sharing, edge-sharing [BO_6_] octahedra form additional chemical bonds between metal sites, enhancing structural stability. Developing efficient strategies to promote stable reconstruction could enrich the toolbox for advanced electrocatalyst design.

In this study, we report distorted RuO_2_ (d-RuO_2_), a monometallic oxide with lattice hydroxyl incorporation, as an active and stable OER electrocatalyst in acidic media. The incorporation of lattice hydroxyl enhances the flexibility of the periodic structure, thereby accelerating the turnover of the Ru oxidation state and promoting the formation of stable edge-sharing [RuO_6_] octahedra during the OER. Additionally, the small particle size of the catalyst decreases the three-phase contact line and facilitates bubble release. The obtained d-RuO_2_ catalyst exhibits a low overpotential of 150 mV and long-term operational stability of 500 h at 10 mA cm^–2^, outperforming many Ru/Ir-based oxides ever reported. A PEMWE instrument using d-RuO_2_ can operate stably for 348 h at 200 mA cm^–2^. This work not only advances the development of high-performance OER catalysts but also paves a way for designing functional materials for a wide range of electrochemical processes.

## 2 Results and Discussion

### 2.1 Structural Characterization

Inspired by the high thermal stability of amorphous RuO_2_·synthesized under hydrothermal conditions, we employed a two-step hydrothermal-calcination method to synthesize distorted RuO_2_ nanoparticles (d-RuO_2_).^30^ Control samples of amorphous RuO_2_ (a-RuO_2_) and rutile RuO_2_ (r-RuO_2_) were prepared by varying the treatment temperature. Thermogravimetric (TG) was first used to measure the changes in all synthesized samples during the heat treatment process. The mass loss below 200 °C corresponds to the removal of physically adsorbed water, while the loss between 200 and 500 °C is attributed to the loss of lattice hydroxyl (2Ru–OH → Ru–O–Ru + H_2_O). As illustrated in Figure 1a, the weight loss in the range of 200–500 °C is about 7.82, 4.42, and 0.41 wt % for a-RuO_2_, d-RuO_2_, and r-RuO_2_, respectively, which corresponds to RuO_*x*_(OH)_0.67_, RuO_*x*_(OH)_0.36_, and RuO_*x*_(OH)_0.03_, respectively. Fourier transform infrared (FT-IR) spectra show a weak peak shoulder at 3222 cm^–1^ (Figure 1b), corresponding to the stretching vibrations of Ru–OH bonds.^31^ The differences in crystallinity among the three RuO_2_-based samples are evident from the X-ray diffraction (XRD) patterns. As shown in Figure 1c, compared to the amorphous a-RuO_2_ that exhibits no distinct diffraction peaks, d-RuO_2_ displays characteristic peaks at 28, 35, and 54 °, which are close to the main peaks of rutile r-RuO_2_, indicating the formation of a rutile structure. Note that the three main Raman peaks corresponding to the rutile RuO_2_ structure (i.e., E_g_, A_1g_, and B_2g_) were clearly observed in a-RuO_2_ (Figure 1d), suggesting the formation of a local RuO_6_ rutile structure within the amorphous phase.^30^ As the temperature increases, the intensity of Raman peaks increases and the full-width at half-maximum of the peaks decreases, demonstrating improved periodicity of the RuO_6_ octahedral arrangement. The chemical and electronic states of O and Ru of the three RuO_2_ samples were characterized by X-ray photoelectron spectroscopy (XPS). The O 1s XPS spectra can be deconvoluted into three peaks: the lattice oxygen (M-O) at 529.2 eV, the oxygen in lattice hydroxyl (M–OH) at 530.9 eV, and the oxygen in physically adsorbed water (H_2_O) at 532.2 eV (Figure 1e and Table S1). The peak area percentage of M–OH, defined as Area_M–OH_/(Area_M–OH_ + Area_M‑O_), decreases in the order of a-RuO_2_ > d-RuO_2_ > r-RuO_2_, which is consistent with the TG results. The binding energies of oxygen species in metal oxides reflect their interactions with metal cations.^32^ The peak at 465.0 eV in Ru 3p_3/2_ XPS spectra is attributed to the binding energy of Ru­(OH)_3_ (Figure 1f).^33^ The Ru^3+^/Ru^4+^ ratios are 0.45, 0.34, and 0.21 for a-RuO_2_, d-RuO_2_, and r-RuO_2_, respectively, indicating that the lattice hydroxyl facilitates the formation of low-valence Ru^3+^ in RuO_2_.

**Figure 1:**
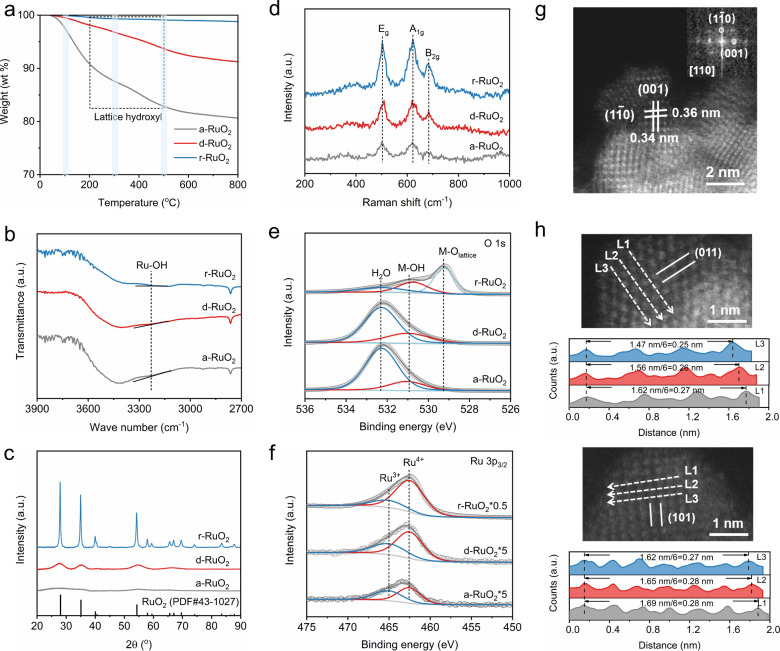
(a) TG curves of a-RuO_2_, d-RuO_2_, and r-RuO_2_. The shading represents the temperature at which the samples are synthesized. (b) FT-IR spectra of a-RuO_2_, d-RuO_2_, and r-RuO_2_. The slope of the line provides a guide to distinguish the intensity of the shoulder corresponding to the Ru–OH bond. (c) XRD patterns of a-RuO_2_, d-RuO_2_, and r-RuO_2_. (d) Raman spectra of a-RuO_2_, d-RuO_2_, and r-RuO_2_. (e) O 1s XPS spectra of a-RuO_2_, d-RuO_2_, and r-RuO_2_. (f) Ru 3p_3/2_ spectra of a-RuO_2_, d-RuO_2_, and r-RuO_2_. (g) HAADF-STEM image of d-RuO_2_. The inset is the Fast Fourier Transform (FFT) image. (h) Enlarged HAADF-STEM images of d-RuO_2_ and measured atomic distance.

High-angle annular dark field scanning transmission electron microscopy (HAADF-STEM) was conducted to analyze the atomic structure of d-RuO_2_. The nanoparticles are uniformly dispersed with a size distribution of 3–4 nm (Figure S1). As depicted in Figure 1g, d-RuO_2_ exhibits a well-defined P42/mnm crystal structure with interplanar distances of 0.36 and 0.34 nm, which are substantially larger than those of the standard rutile RuO_2_ (110) and (001) facets (0.32 and 0.31 nm, respectively). This suggests that lattice hydroxyl induces structural distortion and expands the Ru–Ru distance. The images shown in Figure 1h further confirm the variation of the Ru–Ru distance. Moreover, the electron paramagnetic resonance (EPR) spectrum shows a significant signal at *g*_e_ = 2.003, suggesting abundant oxygen vacancies in the structurally distorted d-RuO_2_ (Figure S2). In contrast, a-RuO_2_ consists of large nanoparticles with an average size of 200 nm and does not display any visible lattice fringes (Figure S3), indicating a high lattice hydroxyl concentration in the amorphous structure. For r-RuO_2_, the particle size is around 20 nm, and the interplanar distance of 0.32 nm agrees with the *d*-spacing of the (110) facet (Figure S4), according to the standard rutile structure. Based on the above results, we can conclude that as the temperature increases, the lattice hydroxyl content decreases, causing the structure to transit from amorphous to distorted and, eventually, to a well-ordered rutile phase. Correspondingly, the particle size decreases from 200 nm (for a-RuO_2_) to 4 nm (for d-RuO_2_) before increasing to 20 nm (for r-RuO_2_).

### 2.2 Performance Evaluation

We measured the electrocatalytic performance of a-RuO_2_, d-RuO_2_, and r-RuO_2_ for the OER in 0.1 M HClO_4_ solution. Linear sweep polarization curves show that d-RuO_2_ achieves a low overpotential of 150 mV to reach an anodic current density of 10 mA cm^–2^ (Figure 2a), suggesting a superior catalytic activity. In comparison, a-RuO_2_ displays comparable activity, while r-RuO_2_ requires a higher overpotential of 220 mV, revealing that lattice hydroxyl is conducive to initiating the OER. To gain a deep understanding of the OER mechanism and kinetics, Tafel plots derived from the polarization curves are shown in Figure 2b. The Tafel slopes of the three samples are around 40 mV dec^–1^, close to the featured Tafel slope of 60 mV dec^–1^, suggesting that the rate-determining step is the O–O bond formation.^34^ The d-RuO_2_ displays the smallest Tafel slope and the fastest reaction kinetics. Considering that mass activity (MA, normalized to noble metal loading) is vital for commercial applications, we compared the value of MA for electrocatalysts at 1.4 V.^35^ The d-RuO_2_ shows a mass activity of 103 A g^–1^, which is 0.4- and 33.3-fold higher than those of a-RuO_2_ (76 A g^–1^) and r-RuO_2_ (3 A g^–1^) (Figures 2c and S5). The structural features of electrocatalysts can affect the permeability of electrolyte and the density of catalytically active sites. The electrochemical active surface area (ECSA) of electrocatalysts was evaluated by double-layer capacitance (*C*_dl_) measurements (Figure S6). The *C*_dl_ value of a-RuO_2_ is 142 mF cm^–2^, which is substantially higher than those of d-RuO_2_ (95 mF cm^–2^) and r-RuO_2_ (11 mF cm^–2^). When the electrocatalytic activity is normalized to the ECSA, a high specific activity of 0.009 mA cm^–2^ is obtained for d-RuO_2_ at 1.4 V, which is 1.3- and 2.0-fold higher than those of a-RuO_2_ (0.004 mA cm^–2^) and r-RuO_2_ (0.003 mA cm^–2^), respectively (Figures 2c and S7). Furthermore, we evaluated the OER activity of RuO_2_ treated at 200 and 400 °C (RuO_2_-200 and RuO_2_-400). The RuO_2_-200 and RuO_2_-400 exhibit inferior activity compared to d-RuO_2_ (Figure S8), highlighting the optimal content of lattice hydroxyl in d-RuO_2_. Notably, the specific activity of d-RuO_2_ is 8 times higher than that of commercial RuO_2_ (com-RuO_2_) (Figure S8), demonstrating the superior OER activity of d-RuO_2_.

**Figure 2:**
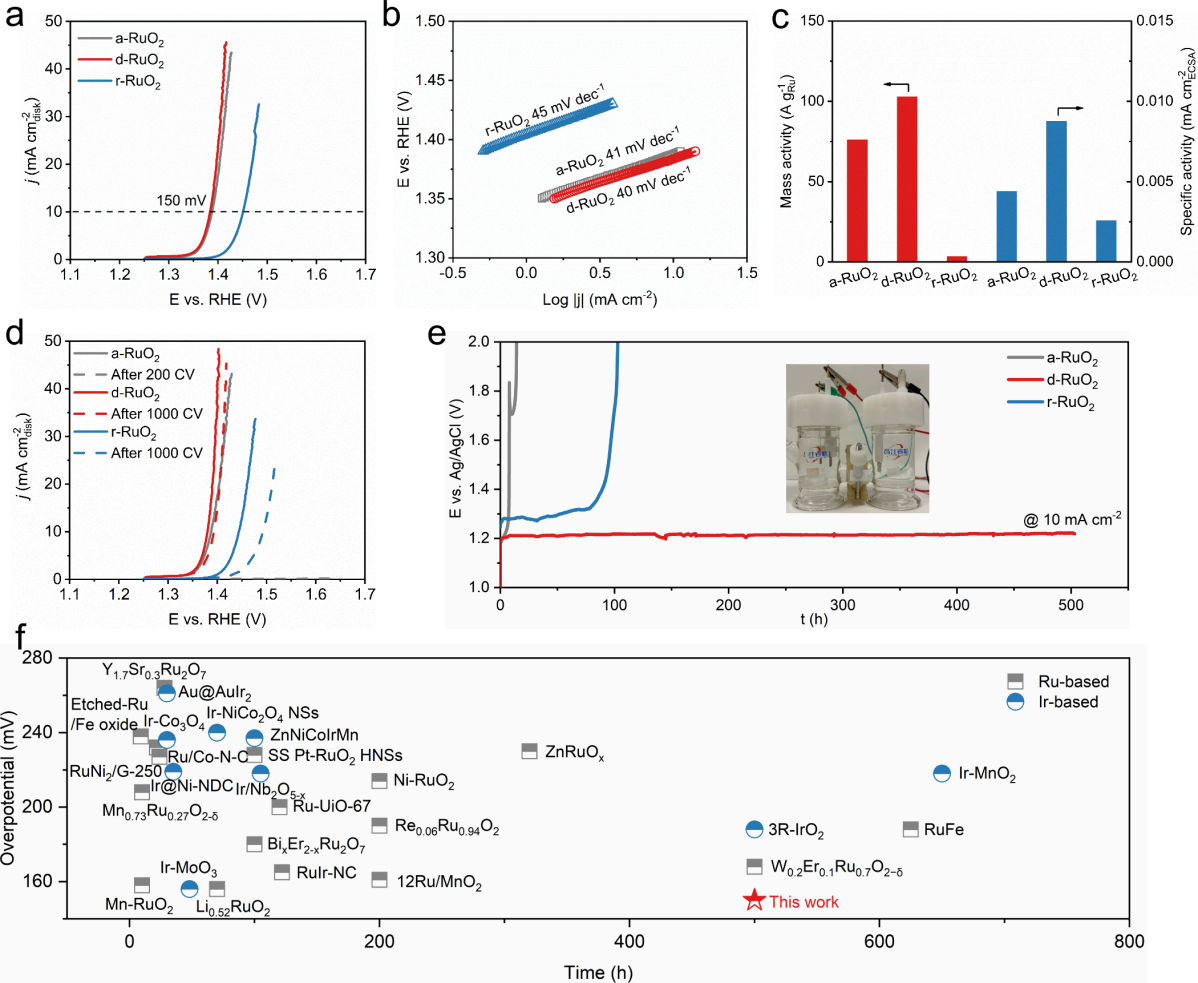
(a) Polarization curves and (b) corresponding Tafel slopes for a-RuO_2_, d-RuO_2_, and r-RuO_2_ electrocatalysts in 0.1 M HClO_4_ electrolyte. (c) Mass activities and specific activities for a-RuO_2_, d-RuO_2_ and r-RuO_2_ at an overpotential of 170 mV. (d) OER polarization curves before and after 1000 continuous cycles for d-RuO_2_ and r-RuO_2_ electrocatalysts, and before and after 200 cycles for a-RuO_2_. (e) Chronopotentiometric responses for a-RuO_2_, d-RuO_2_, and r-RuO_2_ at 10 mA cm^–2^. The inset is a photograph of a H-type electrolytic cell. (f) Comparison of the overpotential and operational stability at 10 mA cm^–2^ of d-RuO_2_ with various Ru- and Ir-based electrocatalysts.

While high OER activity is essential, stability is even more crucial for practical applications under acidic conditions. Initially, a-RuO_2_, d-RuO_2_, and r-RuO_2_ samples loaded on a glass carbon electrode were subjected to continuous cycling for 1000 cycles between 1 and 1.4 V in 0.1 M HClO_4_ solution. As depicted in Figure 2d, the catalytic activity of a-RuO_2_ declines by 100% after 200 cycles, with its *C*_dl_ value decreasing from 153 to 1.1 mF cm^–2^ (Figure S9). To mitigate the deposition of dissolved Ru ions onto the counter electrode, long-term chronoamperometric tests at 10 mA cm^–2^ were performed in a H-type electrolytic cell. Figure 2e shows that a-RuO_2_ experiences a rapid degradation and lasts no more than 15 h, accompanied by significant dissolution of Ru ions (Figure S10). This phenomenon suggests that a-RuO_2_ with an amorphous structure easily transforms into soluble RuO_4_ species under high anodic potentials, leading to structural collapse and the loss of the catalytically active Ru sites, in agreement with other studies.^36^ By contrast, the activity loss of d-RuO_2_ is negligible, with only a 10-mV negative shift and a much smaller loss in the *C*_dl_ value of 0.6% (Figure S9). Furthermore, d-RuO_2_ exhibits a marginal potential increase after 500 h of continuous operation (Figure 2e), and the concentration of dissolved Ru ions is lower than that of a-RuO_2_ (Figure S10), demonstrating the robust stability of d-RuO_2_ with a distorted structure. In the case of r-RuO_2_, the overpotential increases by 50 mV at 10 mA cm^–2^ after 1000 CV tests (Figure 2d), along with a 50% loss in the *C*_dl_ value (Figure S9). The instability of r-RuO_2_ is further confirmed by its rapid deactivation within 103 h during a chronopotentiometric test (Figure 2e). The stability number (S-number) at 1 h follows the order of a-RuO_2_ < d-RuO_2_ < r-RuO_2_ (Table S2). Moreover, the S-number at 5 h is improved due to the dissolution of the defect sites. The S-number of r-RuO_2_ is higher than that of d-RuO_2_, indicating that the structural stability of the electrocatalyst is not the key factor behind the operational instability of r-RuO_2_. We observed large gas bubbles formed on the r-RuO_2_ electrode, resulting in peeling off of the catalyst during the test (Figure S11). The bubbling behavior of a-RuO_2_, d-RuO_2_, and r-RuO_2_ samples will be discussed in detail later. Together, these results suggest that the d-RuO_2_ catalyst has an excellent OER activity and operational stability in a harsh acidic electrochemical environment, which is the top-level catalytic performance among Ru- and Ir-based catalysts reported so far (Figure 2f and Table S3). More importantly, the PEMWE using d-RuO_2_ as an anodic catalyst can operate stably at 200 mA cm^–2^ for 348 h (Figure S12), demonstrating its potential for commercialization.

### 2.3 Mechanism Investigation

To gain insights into the catalytic behavior of d-RuO_2_, in situ X-ray absorption spectroscopy (XAS) was performed to investigate the chemical state and local structure of the active Ru sites during the OER. Figure 3a,b show that the as-prepared d-RuO_2_ exhibits a shift toward lower energy in the Ru K-edge X-ray adsorption near-edge structure (XANES) spectra compared to the control sample r-RuO_2_. This shift indicates a lower Ru oxidation state for d-RuO_2_, in line with XPS results.^37^ The extended X-ray absorption fine structure (EXAFS) data displayed in Figure 3c,d correspond to three kinds of local structures in terms of the apparent distances: the first peak around 1.96 Å is typical for oxygen coordinated Ru ions, the second peak around 3.45 Å is assignable to di-μ-oxo bridged Ru ions (Ru–Ru_edge_), and the third peak around 3.95 Å is attributed to mono-μ-oxo connected Ru ions (Ru–Ru_corner_).^38^ In d-RuO_2_, the Ru–O bond distance is longer than that in r-RuO_2_. This suggests that lattice hydroxyl elongates the Ru–O distance by decreasing the oxidation state of Ru. Correspondingly, longer Ru–Ru_corner_ and Ru–Ru_edge_ distances were observed in d-RuO_2_. Furthermore, d-RuO_2_ shows weaker signals from Ru–Ru_edge_ and Ru–Ru_corner_, whereas these signals are stronger in r-RuO_2_. These data suggest that short-range [RuO_6_] units dominate in d-RuO_2_, whereas a longer-range ordered structure is present in r-RuO_2_.^39^

**Figure 3:**
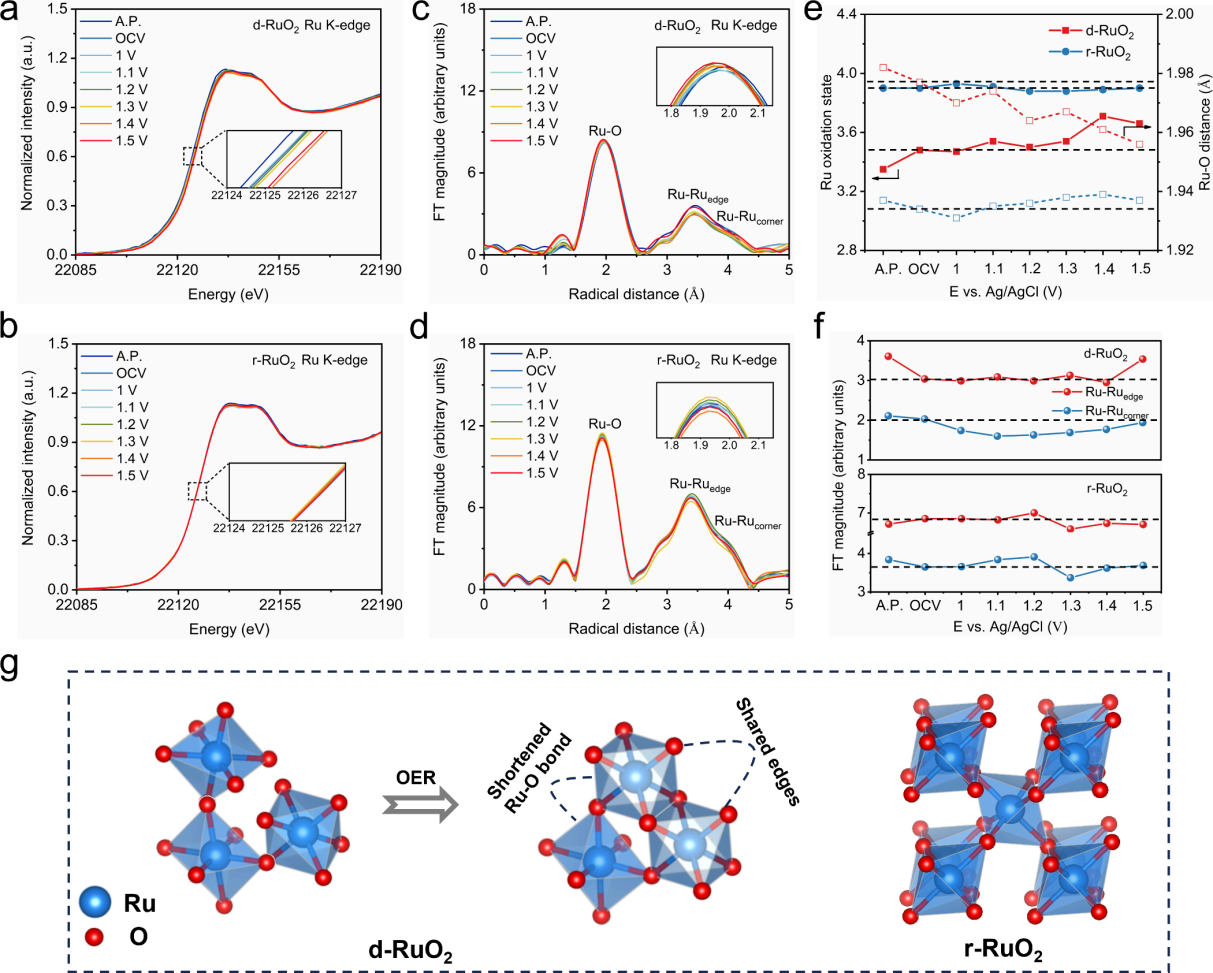
In situ XANES spectra at various bias voltages during the OER for (a) d-RuO_2_ and (b) r-RuO_2_. The insets show an enlarged view of the adsorption edge region. A.P.: as-prepared electrocatalysts. OCV: soaking electrocatalysts in 0.1 M HClO_4_ electrolyte. In situ EXAFS spectra at various bias voltages during the OER for (c) d-RuO_2_ and (d) r-RuO_2_. The insets show an enlarged view of the first shell peak. (e) Oxidation state of Ru and corresponding Ru–O bond distance in d-RuO_2_ and r-RuO_2_ versus control potentials. (f) Magnitude of the Ru–Ru_edge_ and Ru–Ru_corner_ peaks in d-RuO_2_ and r-RuO_2_ versus control potentials. (g) Schematic model of the OER occurring at Ru active sites on the d-RuO_2_ and r-RuO_2_.

Detailed analysis reveals that once immersed in HClO_4_ solution without applied potentials, the oxidation state of Ru in d-RuO_2_ increases from 3.35 to 3.49, accompanied by a shrinkage in the Ru–O bond (Figures 3e, S13, and S14 and Table S4). This suggests that d-RuO_2_ undergoes reconstruction in the electrolyte, which is also observed in the literature.^40^ As the applied potential increases to 1.5 V, the oxidation state of Ru further rises to 3.66 but remains below 4, indicating that the overoxidation of Ru does not occur. Correspondingly, the Ru–O bond length decreased to 1.956 Å. In contrast, the oxidation state of Ru in r-RuO_2_ shows no noticeable change throughout the entire potential sweeping steps, indicating the maintenance of the rutile structure of r-RuO_2_ during the OER. The subtle elongation of the Ru–O bond is attributed to the surface-adsorbed oxygen species.^41^ In rutile r-RuO_2_, [RuO_6_] octahedra are periodically connected, thus making the changes in the Ru oxidation state very difficult, while in distorted d-RuO_2_, the distance between [RuO_6_] octahedra is expanded by lattice hydroxyl; thus, their interactions are much weaker, and redox behavior would become much easier. Recent studies suggest that the oxidation state turnover is closely related to the high activity of OER catalysts.^32,42^ The online differential electrochemical mass spectrometry (DEMS) shows that the OER follows the AEM or the OPM mechanism in our system, rather than the LOM mechanism (Figure S15 and Table S5). Both mechanisms rely on the redox flexibility of metal centers. For AEM, the first elementary reaction step involves the transition of the M–O bond from single to double bonds along with an increase in the metal oxidation state (M^*n*^–OH → M^*n*^ ^+1^=O + H^+^ + e^–^). Subsequently, the bonding state of the M–O bond and the oxidation state of the metal center turn back to their initial states (M^*n*+1^=O + H_2_O → M^*n*^–OOH + H^+^ + e^–^).^43,44^ For OPM, two M=O species are combined to generate O–O bond (2M^*n*+1^=O → M^*n*–1^ + O_2_).^17,18^ Here, variation in the Ru oxidation state in d-RuO_2_ is able to promote the formation of an O–O bond during the OER cycles, thereby effectively accelerating water oxidation. In contrast, the change of the Ru oxidation state is much more difficult in r-RuO_2_ due to its rigid structure; thus, the water oxidation kinetics are hindered on r-RuO_2_. Notably, the amplitude and coordination number of the second-shell Ru–Ru_edge_ peak in d-RuO_2_ increase as the potential increases from the level of OCV to 1.5 V (Figures 3f and S14 and Table S4). Compared to corner-sharing motifs, edge-sharing structures possess one more couple of connected bonds between Ru-site clusters, which improve structural stability and suppress the overoxidation of Ru to soluble RuO_4_.^45^ Based on the above analysis, we can conclude that the flexible structure of active Ru sites in d-RuO_2_ facilitates changes in the oxidation state and promotes the formation of stable edge-sharing RuO_6_ octahedra during the OER (Figure 3g), which improves the catalytic performance.

### 2.4 Bubbling Behavior

For gas evolution reactions (e.g., the OER), the operational stability of catalysts depends not only on their crystal structure but also on their bubbling behavior. First, we investigated the nucleation and growth sites of the gas bubbles. The contact angles of a-RuO_2_, d-RuO_2_, and r-RuO_2_ are all less than 90 ° (Figure 4a). The hydrophilicity allows the electrolyte to fully contact the active sites and reduces the interfacial ohmic resistance. However, the intrinsic hydrophilicity of conductive carbon is usually limited, with a contact angle of 120 °. When a mixture of catalyst and conductive carbon was loaded onto the hydrophobic carbon paper (a-RuO_2_–C/CP, d-RuO_2‑_C/CP, and r-RuO_2_–C/CP), numerous bubbles were observed on the r-RuO_2_–C/CP electrode, whereas a few bubbles formed on the a-RuO_2_–C/CP and d-RuO_2‑_C/CP electrodes (Figure S16). Moreover, we compared electrodes of r-RuO_2_ on hydrophobic carbon paper (r-RuO_2_/CP) and hydrophilic platinum-coated titanium felt (r-RuO_2_/Pt–Ti). The r-RuO_2_/CP electrode shows extensive bubble formation, while the r-RuO_2_/Pt–Ti electrode remains bubble-free (Figure S16). This suggests that bubbles preferentially nucleate and grow on the hydrophobic carbon surface. At the interface between the hydrophilic RuO_2_ and the hydrophobic conductive carbon, the generated bubbles deform, that is, the front contact angle and rear contact angle are different, resulting in Laplace pressure that provides the driving force for the directional movement of the bubbles from RuO_2_ to the conductive carbon.^46^

**Figure 4:**
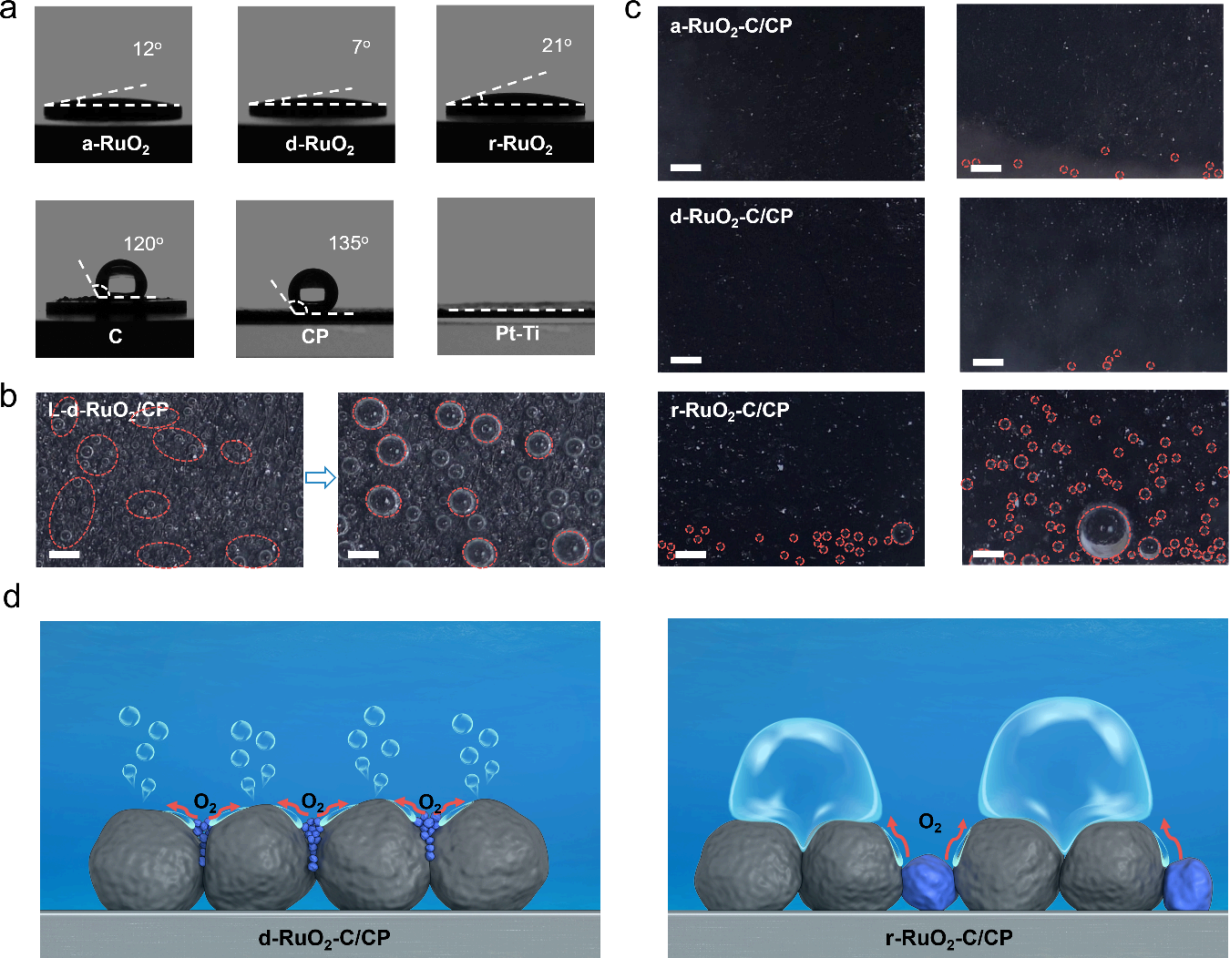
(a) Contact angles of water toward a-RuO_2_, d-RuO_2_, r-RuO_2_, conductive carbon, CP and Pt–Ti. (b) Digital images showing the bubble generation behavior on L-d-RuO_2_/CP electrode at 1 s and 1 min. The OER current density is set at 10 mA cm^–2^. The scale bar is 500 μm. (c) Digital images showing the bubble generation behavior on a-RuO_2_–C/CP, d-RuO_2_–C/CP, and r-RuO_2_–C/CP electrodes at 1 min. The OER current densities on the left and right sides are set at 10 and 50 mA cm^–2^, respectively. The scale bar is 500 μm. (d) Schematics of bubbling behavior on the surfaces of the d-RuO_2_–C/CP and r-RuO_2_–C/CP electrodes.

Next, we investigated the bubbling behavior of d-RuO_2_ loaded on CP with ultralow mass density (L-d-RuO_2_/CP). At 10 mA cm^–2^, bubbles are densely distributed on the surface and grow to ∼500 μm after 1 min (Figure 4b). The CP usually possesses abundant defects, which provide numerous nucleation sites for the bubbles. According to Klausner et al., the component of the surface tension in the horizontal direction is easier to balance the buoyancy than the component in the vertical direction, which ensures that bubbles slide along the carbon fibers.^47^ The fibrous structure of CP further promotes bubble sliding and coalescence. Since hydrophobic CP cannot be completely wetted by the solution, residual gas trapped in pores increases the bubble adhesion force. Consequently, the coalesced bubbles continued to grow instead of detaching from the surface. The L-r-RuO_2_/CP exhibits similar behavior (Figure S17).

At high mass loading, the bubble size of a-RuO_2_–C/CP, d-RuO_2_–C/CP, and r-RuO_2_–C/CP decreases (Figure 4c) because reduced porosity and residual gas weaken the adhesive force. For a-RuO_2_–C/CP and d-RuO_2_–C/CP electrodes, no obvious bubbles formed at 10 mA cm^–2^, while only a few small bubbles (∼110 μm) were observed at 50 mA cm^–2^. The bubble size for the r-RuO_2_–C/CP electrode at 10 and 50 mA cm^–2^ can reach 360 and 860 μm, respectively, which are larger than those for a-RuO_2_–C/CP and d-RuO_2_–C/CP. After the OER measurement, the a-RuO_2_–C/CP electrode becomes more hydrophilic, which may be related to the formation of soluble RuO_4_ species (Figure S18). The d-RuO_2_–C/CP and r-RuO_2_–C/CP electrodes remain hydrophobic. The difference in bubble sizes between d-RuO_2_–C/CP and r-RuO_2_–C/CP can be attributed to the particle size of the catalysts. As illustrated in Figures 4d and S19, the dense distribution of hydrophilic d-RuO_2_ particles in the gaps between carbon particles decreases the three-phase contact line, thereby lowering the adhesion force, which is confirmed by the lower contact angle (122°, Figure S18). The small adhesion force can be quickly overcome by the Laplace pressure and buoyancy generated during bubble coalescence, leading to smaller bubbles left.^48^ In contrast, carbon particle aggregation in r-RuO_2_–C/CP not only facilitates bubble nucleation but also increases the three-phase contact line, thereby increasing the number of bubbles and the adhesion force, as evidenced by the higher contact angle (131°, Figure S18). Consequently, numerous bubbles form, coalesce, and grow until the buoyancy exceeds the adhesion force. This prolonged residence of bubbles induces a nonuniform current distribution on the electrocatalyst surface, and the drag forces generated by bubble detachment can seriously damage the catalyst layer, thereby accelerating electrocatalyst degradation (Figure S20).

## 3 Conclusions

In summary, monometallic RuO_2_ (d-RuO_2_) was developed as an active and stable OER electrocatalyst via a lattice hydroxyl incorporation strategy. Experimental results suggest that lattice hydroxyl elongates the Ru–Ru distance and induces structural distortion. Consequently, the flexible structure of d-RuO_2_ promotes the change of the Ru oxidation state and the formation of stable edge-sharing [RuO_6_] octahedra during the OER, which enhances the catalytic activity and mitigates the Ru dissolution process. Furthermore, the small d-RuO_2_ nanoparticles are densely distributed on conducive carbon supports, which decreases the three-phase contact line and reduces the bubble size. As a result, the d-RuO_2_ catalyst exhibits superior performance and durability owing to rapid Ru oxidation state turnover, stable structural rearrangement, and efficient bubble management. This study not only sheds light on a fundamental understanding of the OER process on distorted RuO_2_ but also paves an avenue for the development of efficient catalysts for various electrochemical reactions.

## 4 Experimental Section

### 4.1 Synthesis of d-RuO_2_ Catalyst

0.1 g portion of RuCl_3_ was dissolved in 25 mL of deionized water and stirred for 30 min. The mixture was then transferred to a 50 mL Teflon-lined autoclave and heated at 100 °C for 24 h to promote the hydrolysis reaction. The amorphous RuO_2_ precursor was rinsed several times with deionized water and dried at 80 °C for 5 h in a vacuum oven. The d-RuO_2_ sample was obtained through calcinating the precursor at 300 °C for 2 h in air. The a-RuO_2_ and r-RuO_2_ samples were prepared under different calcination temperatures (100 and 500 °C), with other processing parameters the same as those of d-RuO_2_.

### 4.2 Electrochemical Measurements

In a typical procedure, 2.5 mg of conductive carbon (Super P Li) and 5 mg of catalysts were dispersed in 388 μL of deionized water, 76 μL of isopropanol, and 36 μL of Nafion solution (5 wt %) by sonication for 30 min. Six μL of well-dispersed catalyst ink was drop-cast on a glassy carbon rotating disk electrode (RDE, Pine, 5 mm in diameter), resulting in a mass loading of 0.306 mg cm^–2^. All RDE measurements were conducted in a standard three-electrode cell equipped with an electrochemical workstation (CHI 760) in a 0.1 M HClO_4_ electrolyte. The catalyst-modified RDE, an Ag/AgCl electrode (3.5 M KCl), and a Pt foil served as the working, reference, and counter electrodes, respectively. The measured potential was *iR* compensated and converted to the RHE scale using *E* (vs RHE) = *E* (vs Ag/AgCl) + 0.25 V + *iR*, where *i* is the current and *R* is the uncompensated resistance. Polarization curves were recorded from 1.0 to 1.4 V (vs Ag/AgCl) at a scan rate of 5 mV s^–1^. The electrochemical surface area (ECSA) was determined by double-layer capacitance (*C*_dl_) according to ECSA = *C*_dl_/*C*_s_, where *C*_s_ is the general specific capacitance (0.035 mF cm^–2^). The *C*_dl_ values were obtained through cyclic voltammetry (CV) tests in the nonfaradaic potential region (0.95–1.05 V vs Ag/AgCl) at different scan rates (2.5, 5, 7.5, and 10 mV s^–1^). Stability was examined by 1000-cycle CV measurements from 1.0 to 1.4 V vs Ag/AgCl, and a chronopotentiometry test was conducted at 10 mA cm^–2^.

For the PEM electrolyzer, the anode catalyst d-RuO_2_ and cathode catalyst Pt/C (60 wt % Pt) were sprayed onto the surface of Nafion 115 with loading of 3 mg_oxide_ cm^–2^ and 1 mg_Pt_ cm^–2^, respectively. Pt-plated Ti fiber felt and carbon paper served as the anode and cathode gas diffusion layers, respectively. The stability of the PEM electrolyzer was evaluated at 200 mA cm^–2^ in 0.1 M HClO_4_.

## Supplementary Material



## Notes

Characterization method; TEM analysis for a-RuO_2_, d-RuO_2_, and r-RuO_2_; EPR spectrum for d-RuO_2_; double-layer capacitance tests for a-RuO_2_, d-RuO_2_, and r-RuO_2_; full-cell water electrolysis experiment; Ru dissolution experiments for a-RuO_2_, d-RuO_2_, and r-RuO_2_; DEMS signals for a-RuO_2_, d-RuO_2_, and r-RuO_2_; optical images for a-RuO_2_–C/CP, d-RuO_2_–C/CP, and r-RuO_2_–C/CP electrodes after stability test; bubbling behavior for L-r-RuO_2_/CP; and contact angle measurements for a-RuO_2_–C/CP, d-RuO_2_–C/CP, and r-RuO_2_–C/CP electrodes before and after stability test (PDF)
