# Supplementary material for: Regulating Ru–Ru Distance in RuO2 Catalyst by Lattice Hydroxyl for Efficient Water Oxidation
Source: ACS Nano. 2025 May 6;19(19):18513–21. doi: 10.1021/acsnano.5c01937 (PMC12096437; doi:10.1021/acsnano.5c01937)
Supplement: Supplementary file 1 [file nn5c01937_si_001.pdf]

Supplementary Information for

Regulating Ru-Ru distance in RuO_2 catalyst by lattice hydroxyl for efficient water oxidation

Sixuan She‡, [1], Hsiao-Chien Chen‡, [2, 3], Changsheng Chen[1], Yanping Zhu[1], Gao Chen[4], Yufei Song[1], Yiping Xiao[1], Zezhou Lin[1], Di Zu[5], Luwei Peng[1], Hao Li[1], Ye Zhu[1], Yuen Hong Tsang, [1, 6, 7] and Haitao Huang*, [1]*

[1]Department of Applied Physics, The Hong Kong Polytechnic University, Hung Hom, Kowloon, Hong Kong, China
[2]Center for Reliability Science and Technologies, Chang Gung University, Taoyuan 33302, Taiwan
[3]Kidney Research Center, Department of Nephrology, Chang Gung Memorial Hospital Linkou, Taoyuan 33305, Taiwan
[4]Jiangsu Key Laboratory of New Energy Devices and Interface Science, School of Chemistry and Materials Science, Nanjing University of Information Science and Technology, Nanjing, 210044, China
[5]School of Materials Science and Engineering, Beijing Institute of Technology, Beijing 100081, China
[6]Photonics Research Institute, The Hong Kong Polytechnic University, Hung Hom, Kowloon, Hong Kong, China
[7]Research Institute for Advanced Manufacturing, The Hong Kong Polytechnic University, Hung Hom, Kowloon, Hong Kong, China

This file includes:

1. Methods
2. Supplementary Figures
3. Supplementary Tables

1. Methods

Characterization. The mass loss of the materials was acquired by Thermogravimetry (TG, Netzsch STA449F5). The sample was calcinated from room temperature to 800 ^{o}C in air at a heating rate of 10 ^{o}C min^{-1}. Fourier transform infrared (FT-IR) spectra of the catalysts pressed into KBr discs were recorded using Bruker vertex 70. The crystallinity of the materials was determined by powder X-ray diffraction (XRD, Rigaku Smartlab with Cu kα radiation). Raman measurements were conducted to analyze the local structure using inVia™ Inspect confocal Raman miscroscope (Renishaw). The surface electronic state was estimated by X-ray photoelectron spectroscopy (XPS, Thermo ESCALAB 250xi). The morphology and structure observations were performed on a JEOL JEM-2100F microscope operated at 200 kV. The scanning transmission electron microscopy (STEM) was performed on a ThermoFisher Scientific Spectra 300 equipped with an X-FEG/UltiMono electron source, a GIF Continuum K3 System and a CEOS SCORR fifth-order probe corrector, operated at 300 kV. Electron paramagnetic resonance (EPR) was carried out on a Bruker A300 at room temperature. In situ X-ray absorption spectra at Ru K-edge were recorded at TLS 44A of NSRRC in Taiwan. The amount of dissolved metal ions in solution was analyzed by Inductively coupled plasma mass spectrometry (ICP-MS, Perkin Elmer NexION 2000). The online differential electrochemical mass spectrometry (DEMS) tests were performed using a HIDEN HPR-20 OEMS device. The bubbling behavior on the electrode surface (0.2 mg cm^{-2} and 3 mg cm^{-2}) was captured using Sony α7c with APS-C 40mm F2.8 MACRO.

2. Supplementary Figures

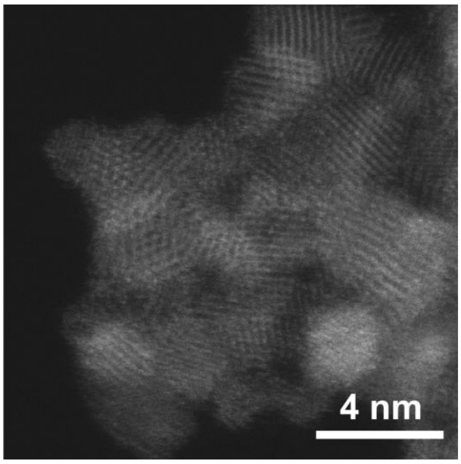

Figure S1. STEM image of d-RuO_2.

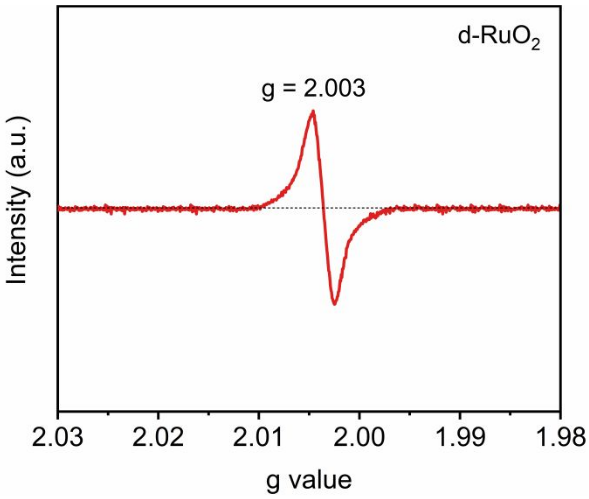

Figure S2. EPR spectrum of d-RuO_2.

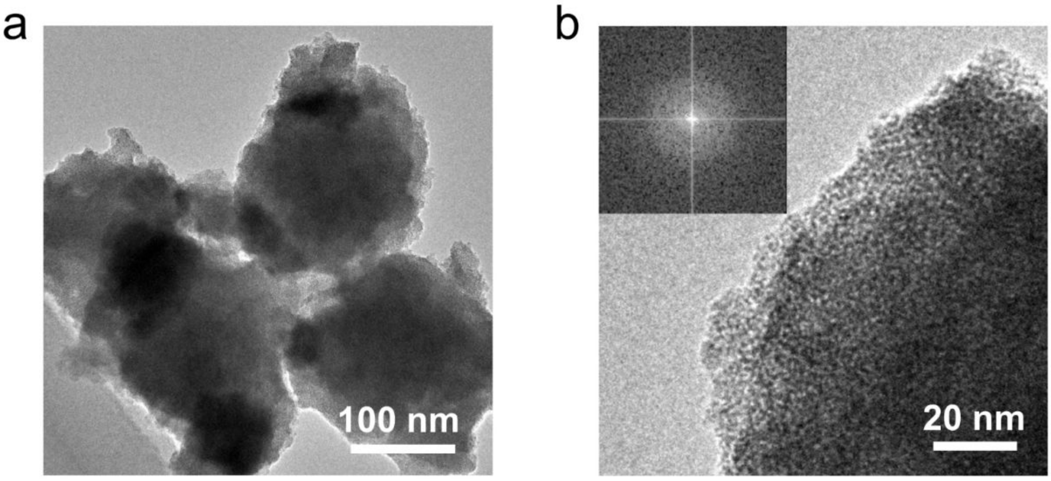

Figure S3. (a) TEM and (b) HR-TEM images of a-RuO_2. The inset is FFT image.

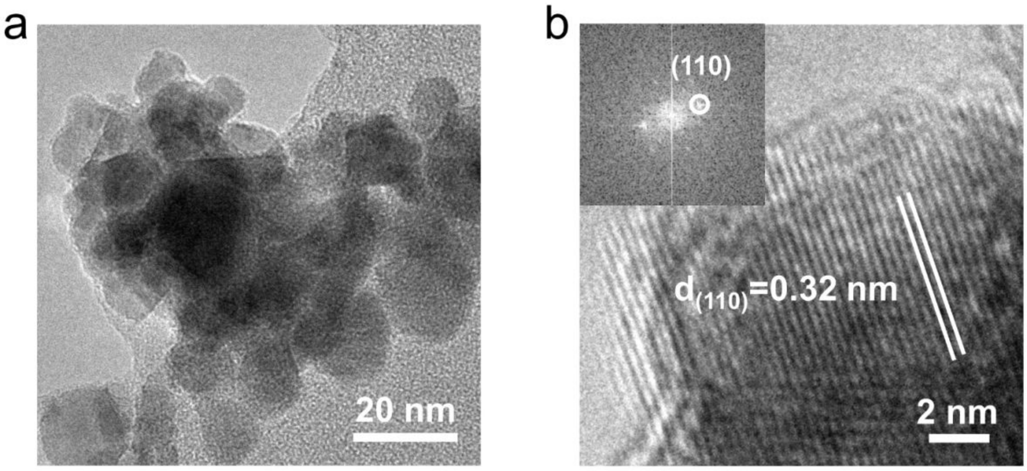

Figure S4. (a) TEM and (b) HR-TEM images of r-RuO_2. The inset is FFT image.

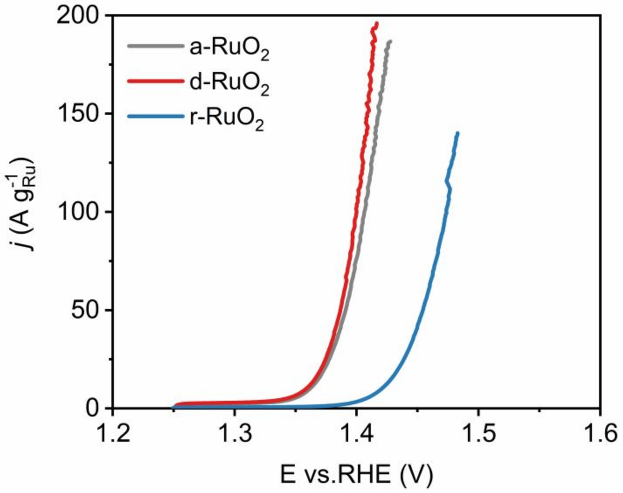

Figure S5. Mass specific polarization curves for a-RuO_2, d-RuO_2 and r-RuO_2 electrocatalysts.

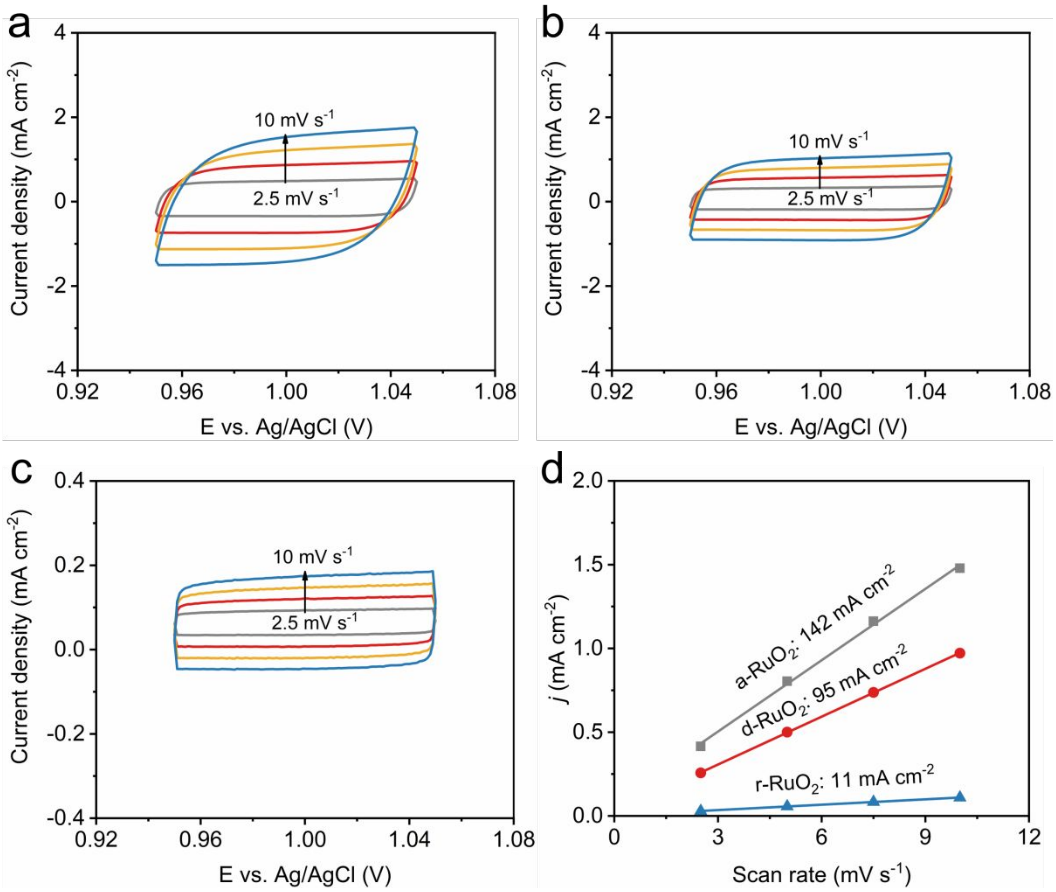

Figure S6. Electrochemical CV curves under different scan rates (2.5, 5, 7.5 and 10 mV s^{-1}) for (a) a-RuO_2, (b) d-RuO_2 and (c) r-RuO_2. (d) Capacitive current densities at 1 V vs. Ag/AgCl as a function of scan rate for a-RuO_2, d-RuO_2 and r-RuO_2 samples, and the corresponding C_{dl} values estimated through linear fitting of the plots.

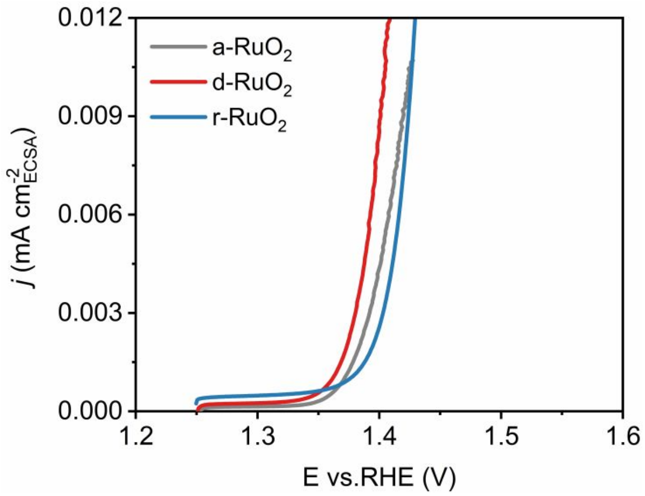

Figure S7. ECSA specific polarization curves for a-RuO_2, d-RuO_2 and r-RuO_2 electrocatalysts.

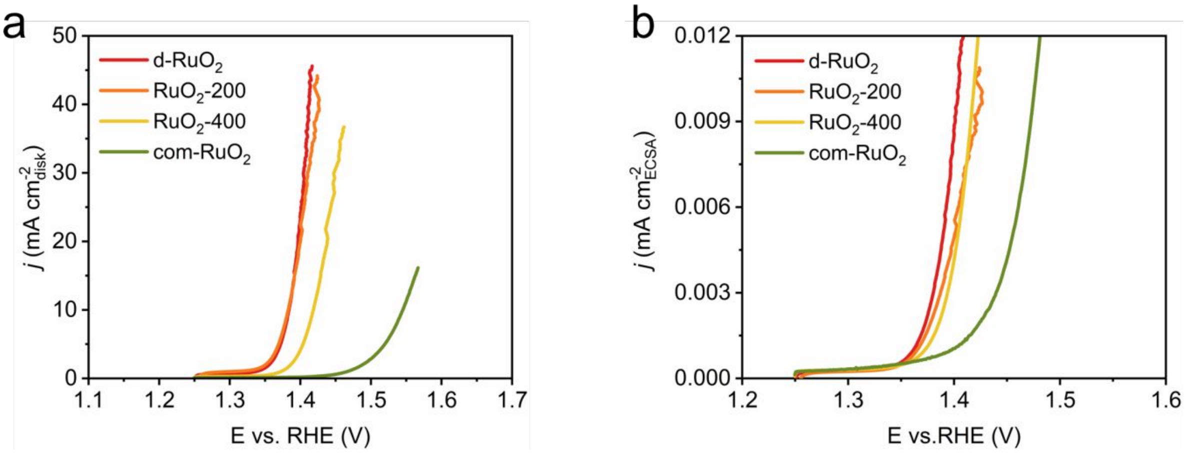

Figure S8. (a) Polarization curves and (b) ECSA specific polarization curves for d-RuO_2, RuO_2-200, RuO_2-400 and com-RuO_2 catalysts.

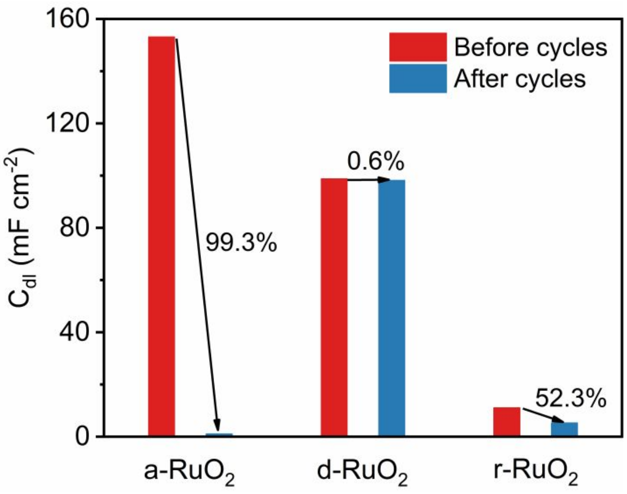

Figure S9. The C_{dl} values before and after 1000 continuous cycles for d-RuO_2 and r-RuO_2 electrocatalysts, and before and after 200 cycles for a-RuO_2.

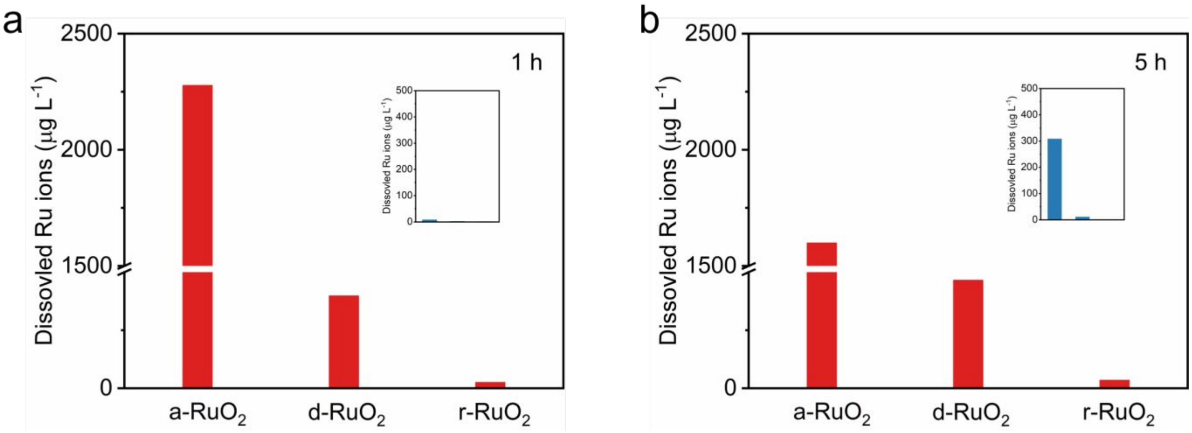

Figure S10. (a) The concentration of dissolved Ru ions in the working electrode chamber after 1-h stability test for a-RuO_2, d-RuO_2 and r-RuO_2 electrocatalysts. (b) The concentration of dissolved Ru ions in the working electrode chamber after 5-h stability test for a-RuO_2, d-RuO_2 and r-RuO_2 electrocatalysts. The insets show the concentration of dissolved Ru ions in the counter electrode chamber.

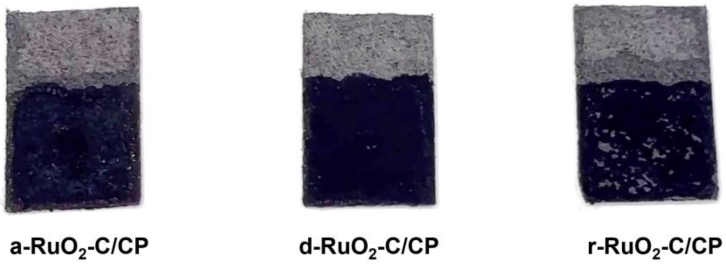

Figure S11. The optical images of a-RuO_2-C/CP, d-RuO_2-C/CP and r-RuO_2-C/CP electrodes after long-term stability measurement.

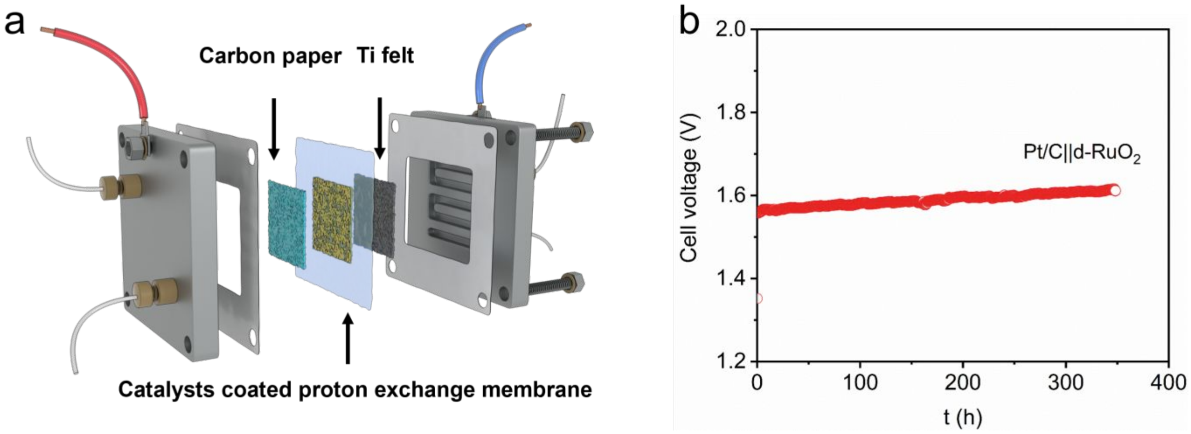

Figure S12. (a) Schematic diagram of the PEM electrolyzer. (b) Chronopotentiometry test of d-RuO_2 catalyst at 200 mA cm^{-2} in the PEM electrolyzer measured at 80 ^{o}C.

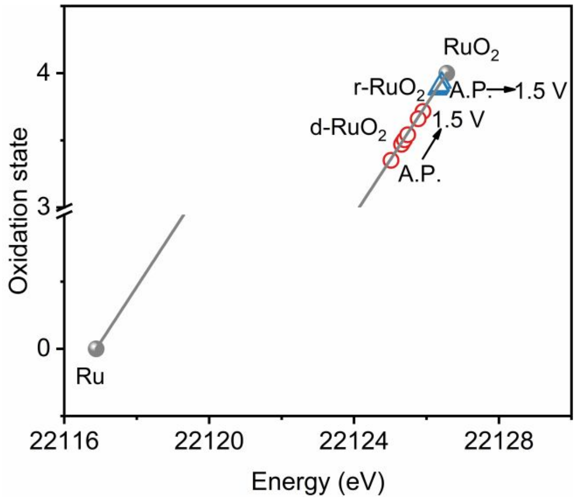

Figure S13. The estimated valence of Ru for d-RuO_2 and r-RuO_2 electrocatalysts during the OER at different potentials.

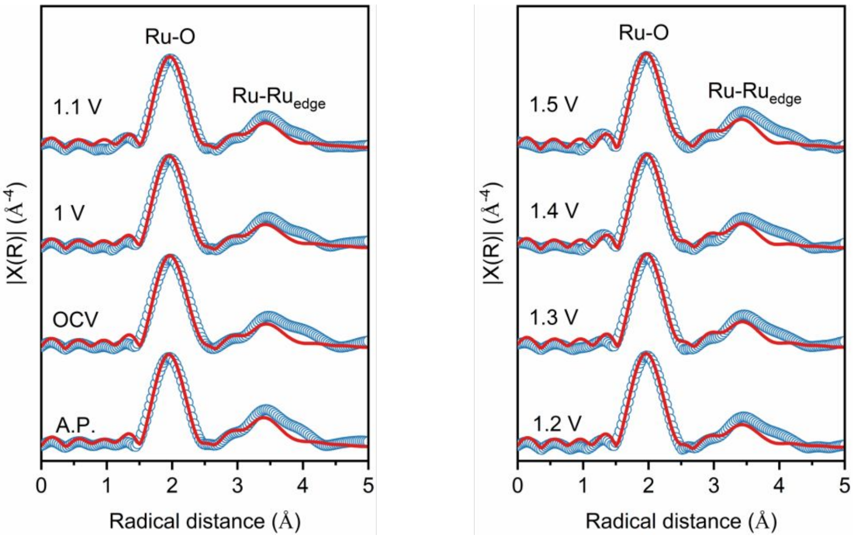

Figure S14. Fourier transform of Ru K-edge EXAFS spectra (hollow circles) at various bias voltages and the corresponding fitting results (solid lines) for d-RuO_2.

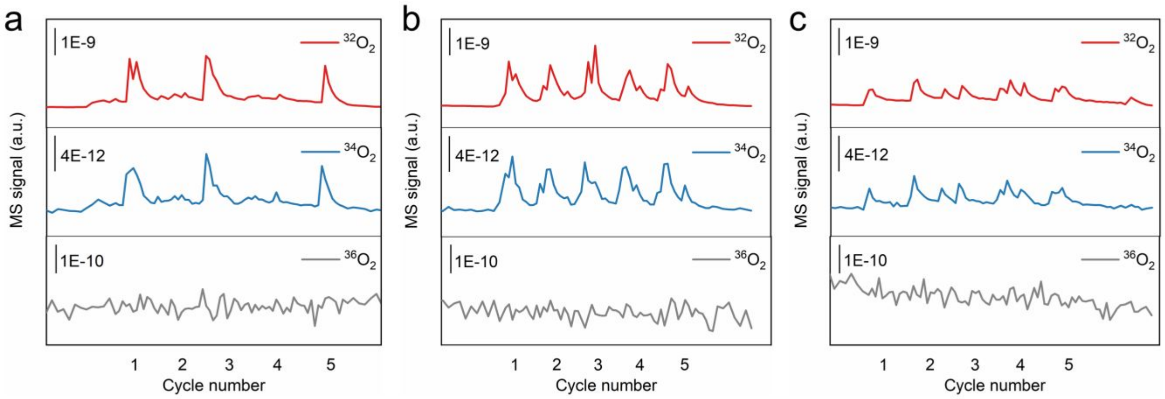

Figure S15. DEMS signal of O_2 products for ^{18}O-labed (a) a-RuO_2, (b) d-RuO_2 and (c) r-RuO_2 in 0.1 M $HClO_4$ with $H_2{}^{16}O$ solvent.

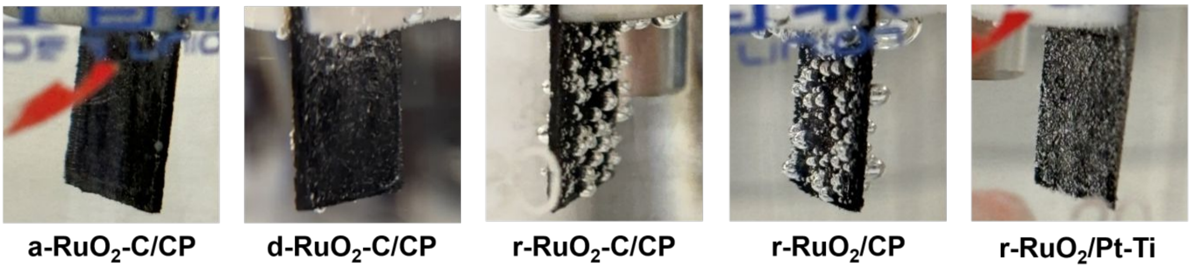

Figure S16. The photographs of a-RuO_2-C/CP, d-RuO_2-C/CP, r-RuO_2-C/CP, r-RuO_2/CP and r-RuO_2/Pt-Ti electrodes.

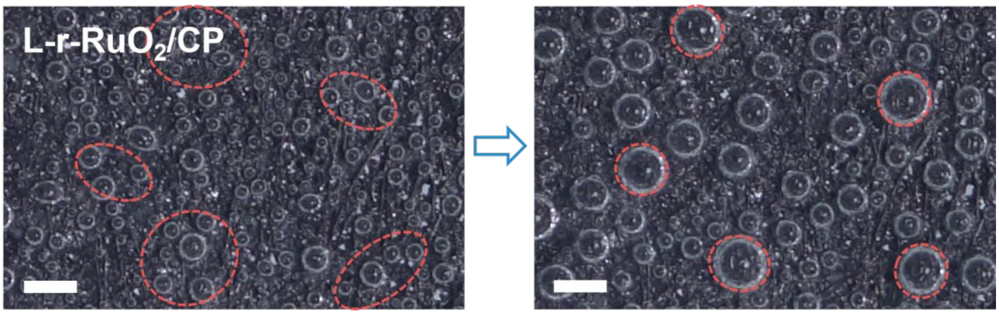

Figure S17. Digital images showing the bubble generation behavior on L-r-RuO_2/CP electrode at 1 s and 1 min. The OER current density is set at 10 mA cm^{-2}. The scale bar is 500 μm.

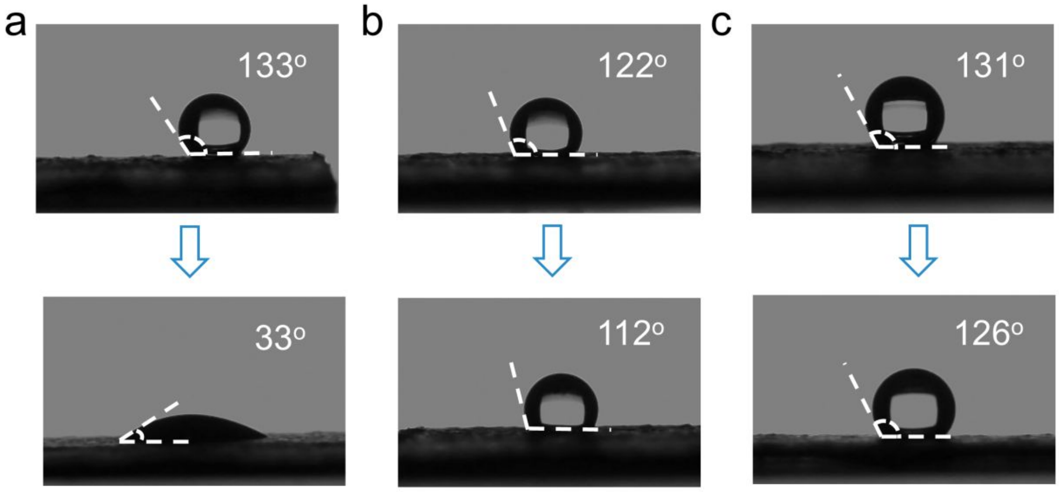

Figure S18. Contact angles of (a) a-RuO_2-C/CP, (b) d-RuO_2-C/CP and (c) r-RuO_2-C/CP electrodes before and after OER measurements.

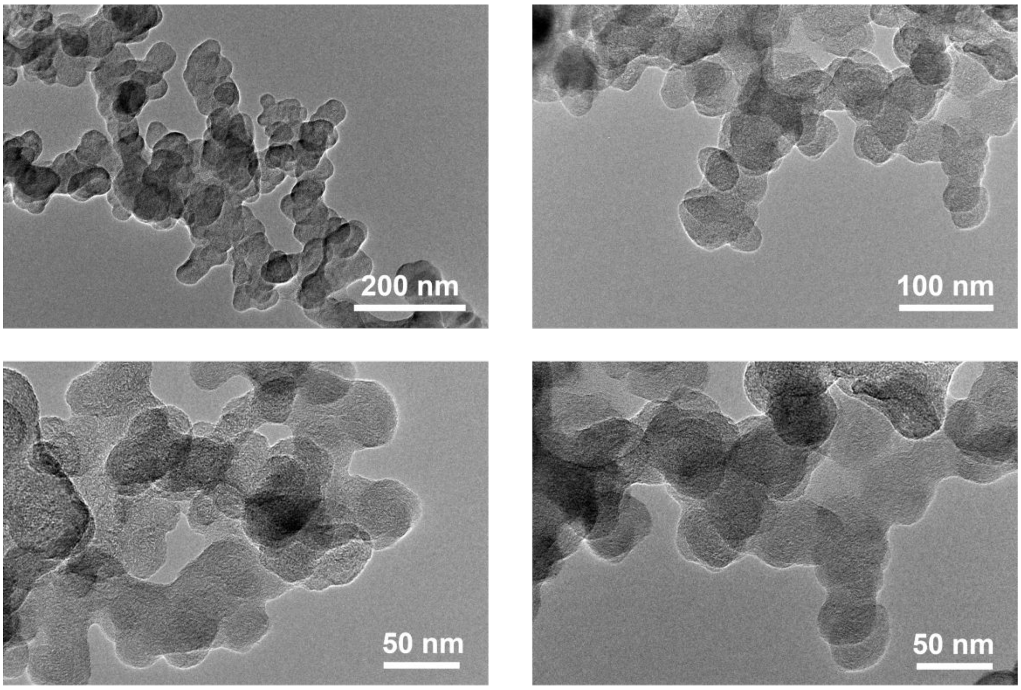

Figure S19. TEM images of conductive carbon.

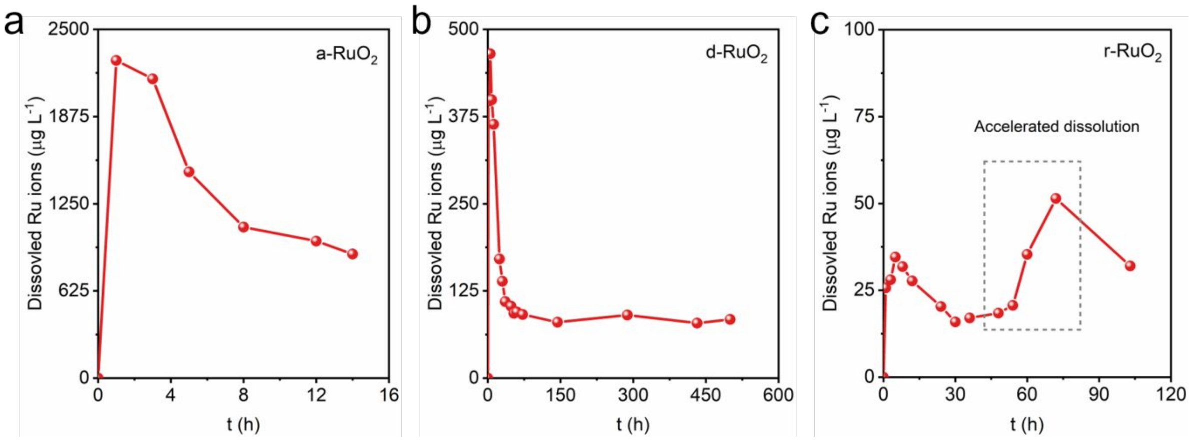

Figure S20. The amount of dissolved Ru ions for (a) a-RuO_2-C/CP, (b) d-RuO_2-C/CP and (c) r-RuO_2-C/CP electrodes.

3. Supplementary Tables

Table S1. The peak area of the three different surface oxygen species for a-RuO_2, d-RuO_2 and r-RuO_2 samples.

Samples	M-O	M-OH	H_2O
a-RuO_2	364478.8	219178.1	156145.6
d-RuO_2	76428.21	304589.2	829504.7
r-RuO_2	21.58225	232637.8	958316.9

Table S2 The S-number of a-RuO_2, d-RuO_2 and r-RuO_2 catalysts at 1 h and 5 h.

Samples	S-number (1 h)	S-number (5 h)
	n_{O2}/n_{Ru}	n_{O2}/n_{Ru}
a-RuO_2	34	-
d-RuO_2	198	4690
r-RuO_2	3059	34912

Table S3. Comparison of OER performance for d-RuO_2 and the state-of-the-art Ir and Ru-based electrocatalysts reported in the literatures.

**Catalyst**	**electrolyte**	**η[a] (mV)**	**Stability[b] (h)**	**Ref.**
**d-RuO_2**	**0.1 M $HClO_4$**	**150**	**500**	**This work**
$ZnRuO_x$	0.5 M H_2SO_4	230	320	J. Am. Chem. Soc. 2024, 126, 15515
RuFe	0.5 M H_2SO_4	188	625	Adv. Mater. 2024, 2312369
Ni-RuO_2	0.1 M $HClO_4$	214	200	Nat. Mater. 2023, 22, 100
$Re_{0.06}Ru_{0.94}O_2$	0.1 M $HClO_4$	190	200	Nat. Commun. 2023, 14, 354
Ru-UiO-67	0.5 M H_2SO_4	200	120	Chem 2023, 9, 1882
$Bi_xEr_{2-x}Ru_2O_7$	0.1 M $HClO_4$	180	100	Nat. Commun. 2022, 13, 4106
$Li_{0.52}RuO_2$	0.5 M H_2SO_4	156	70	Nat. Commun. 2022, 13, 3784

SS Pt-RuO_2 HNSs	0.5 M H_2SO_4	228	100	Sci. Adv. 2022, 8, eabl9271
$Mn_{0.73}Ru_{0.27}O_{2-\delta}$	0.5 M H_2SO_4	208	10	Energy Environ. Sci. 2022, 15, 2356
Ru/Co-N-C	0.5 M H_2SO_4	232	22	Adv. Mater. 2022, 34, 2110103
12Ru/MnO_2	0.5 M H_2SO_4	161	200	Nat. Catal. 2021, 4, 1012
$Y_{1.7}Sr_{0.3}Ru_2O_7$	0.5 M H_2SO_4	264	28	ACS Nano, 2021, 15, 8537
Etched-Ru/ Fe oxide	0.5 M H_2SO_4	238	9	Nano Energy 2021, 84, 105909
$W_{0.2}Er_{0.1}Ru_{0.7}O_{2-\delta}$	0.5 M H_2SO_4	168	500	Nat. Commun. 2020, 11, 5368
$RuNi_2$/G-250	0.5 M H_2SO_4	227	24	Adv. Mater. 2020, 32, 1908126
Mn-RuO_2	0.5 M H_2SO_4	158	10	ACS Catal. 2020, 10, 1152
RuIr-NC	0.05 M H_2SO_4	165	122	Nat. Commun. 2021, 12, 1145
ZnNiCoIrMn	0.1 M $HClO_4$	237	100	Adv. Mater. 2023, 35, 2300091
Ir@Ni-NDC	0.5 M H_2SO_4	219	35	Angew. Chem. Int. Ed. 2023, 62, e202302220
Ir-Co_3O_4	0.5 M H_2SO_4	236	30	Nat. Commun. 2022, 13, 7754
Ir/Nb_2O_{5-x}	0.5 M H_2SO_4	218	105	Angew. Chem. Int. Ed. 2022, 61, e202212341
Ir-MoO_3	0.5 M H_2SO_4	156	48	Nat. Commun. 2021, 12, 5676
3R-IrO_2	0.1 M $HClO_4$	188	500	Joule 2021, 5, 3221
Ir-MnO_2	0.5 M H_2SO_4	218	650	Joule 2021, 5, 2164
Au@$AuIr_2$	0.5 M H_2SO_4	261	30	J. Am. Chem. Soc. 2021, 143, 4639
Ir-$NiCo_2O_4$ NSs	0.5 M H_2SO_4	240	70	J. Am. Chem. Soc. 2020, 142, 18378

[a]: Overpotential at the current density of 10 mA cm^{-2};

[b] Chronopotentiometric stability test at the current density of 10 mA cm^{-2}

Table S4 Fitting results of Ru K-edge EXAFS data at various bias voltages for the d-RuO_2 catalyst.

Samples	Bond type	CN	R (Å)	σ^2 ($10^{-3}\times Å^2$)	ΔE_0 (eV)	R* factor
A.P.	Ru-O	5.14(0.58)	1.982(0.040)	2.69(1.22)	-0.21(1.36)	0.0057
	$Ru\text{-}Ru_{edge}$	**1.74(0.26)**	3.456(0.051)	1.09(1.08)		
OCV	Ru-O	5.30(0.76)	1.977(0.044)	3.09(1.60)	1.57(1.70)	0.010
	$Ru\text{-}Ru_{edge}$	**1.64(0.36)**	3.475(0.055)	1.76(1.69)		
1 V	Ru-O	5.04(0.60)	1.970(0.037)	2.48(1.29)	0.68(1.43)	0.0071
	$Ru\text{-}Ru_{edge}$	**1.56(0.27)**	3.460(0.047)	1.53(1.33)		
1.1 V	Ru-O	4.95(0.61)	1.974(0.042)	2.56(1.33)	1.44(1.45)	0.0081
	$Ru\text{-}Ru_{edge}$	**1.57(0.27)**	3.427(0.049)	1.51(1.31)		
1.2 V	Ru-O	5.15(0.70)	1.964(0.036)	2.88(1.50)	2.00(1.61)	0.010
	$Ru\text{-}Ru_{edge}$	**1.58(0.34)**	3.445(0.045)	2.39(1.70)		
1.3 V	Ru-O	5.30(0.88)	1.967(0.045)	3.49(1.85)	3.03(1.84)	0.020
	$Ru\text{-}Ru_{edge}$	**1.62(0.34)**	3.439(0.053)	1.92(1.66)		
1.4 V	Ru-O	5.45(0.75)	1.961(0.040)	4.19(1.57)	3.41(1.52)	0.015
	$Ru\text{-}Ru_{edge}$	**1.61(0.31)**	3.445(0.045)	2.86(1.51)		
1.5 V	Ru-O	5.74(0.91)	1.956(0.034)	4.64(1.85)	3.77(1.79)	0.016
	$Ru\text{-}Ru_{edge}$	**1.74(0.35)**	3.439(0.037)	1.68(1.54)		

Table S5. The $^{34}O_2/(^{32}O_2+^{34}O_2)$ ratios in a-RuO_2, d-RuO_2, and r-RuO_2 as determined by DEMS.

Samples	Peak area of $^{32}O_2$	Peak area of $^{34}O_2$	$^{34}O_2/(^{32}O_2+^{34}O_2)$ ratio (%)
a-RuO_2	3.48E-04	1.15E-06	0.33
d-RuO_2	4.05E-04	1.35E-06	0.33
r-RuO_2	2.57E-04	6.88E-07	0.27
